# Non-invasive neuromodulation for the treatment of drug-resistant epilepsy: Protocol for a systematic review and meta-analysis investigating efficacy, safety, and optimal stimulation parameters

**DOI:** 10.1186/s13643-025-02981-2

**Published:** 2025-11-06

**Authors:** Sayumi Premaratne, Maryam Zoghi, Ana Antonic-Baker, Zhibin Chen, Leo Chen, Ryan Hamer, Brendan Major, Elizabeth H. X. Thomas, Patrick Kwan, Terence J. O’Brien, Brian N. Lundstrom, Hugh D. Simpson

**Affiliations:** 1 Monash University, Melbourne, Australia; 2 Federation University, Melbourne, Australia; 3 Alfred Health, Melbourne, Australia; 4 St. Vincent’s Hospital, Melbourne, Australia; 5 University of Sydney, Sydney, Australia; 6 Mayo Clinic, Rochester, MN USA; 7 University of Melbourne, Melbourne, Australia

**Keywords:** Epilepsy, Drug-resistant Epilepsy, Seizure, Neuromodulation, Non-invasive

## Abstract

**Background:**

Non-invasive neuromodulation presents as an exciting potential adjunctive therapy for people with drug-resistant epilepsy (DRE). A major advantage of this approach is the absence of the neurocognitive and systemic adverse events commonly associated with anti-seizure medications (ASM), and the significant risks that accompany both resective epilepsy surgery and invasive neuromodulation techniques. The proposed systematic review and meta-analysis will investigate the efficacy of repetitive transcranial magnetic stimulation (rTMS), transcranial direct current stimulation (tDCS), transcranial alternating current stimulation (tACS), low-intensity focused ultrasound (LI-FUS), transcutaneous vagus nerve stimulation (tVNS), and trigeminal nerve stimulation (TNS) for seizure reduction amongst patients diagnosed with DRE, with comparisons also being made between intervention types where applicable. Both immediate and long-term adverse events will also be discussed to offer greater insight into its use as a safe, viable, and reliable method of seizure control. The study’s secondary aim will be to identify optimal stimulation parameters to better inform future clinical trial protocols and to maximise treatment efficacy in clinical applications.

**Methods:**

PubMed, OVID Embase, OVID Medline, and Web of Science will be searched for studies investigating the efficacy and safety of non-invasive nerve and brain stimulation techniques for the management of DRE. Two authors will independently screen relevant studies in Covidence, extract data, and assess risks of bias, with discrepancies being resolved by a third reviewer. The primary outcome will be seizure reduction, measured by mean/median change in seizure frequency and responder rate. Secondary outcomes will include the percentage of patients reporting specified adverse events. A meta-analysis will assess the primary outcome. Subgroup analyses will be performed to investigate potential sources of heterogeneity and the optimal protocol settings for each intervention. Sensitivity analyses will also be conducted to evaluate the robustness of the results.

**Discussion:**

This study will analyse all relevant non-invasive brain and nerve stimulation methods using a consistent protocol, uniquely allowing for rigorous comparisons and result-pooling to evaluate the overall efficacy and safety of each stimulation method. Additionally, it will identify optimal stimulation parameters, where possible, to facilitate the use of these methods in future trials and clinical applications.

**Systematic review registration:**

PROSPERO CRD42023446051.

**Supplementary Information:**

The online version contains supplementary material available at 10.1186/s13643-025-02981-2.

## Background

People with epilepsy experience unprovoked seizures, which can be disabling, dangerous, and potentially life-threatening. Seizures arise from the abnormal synchronous firing of electrical impulses within the brain and may originate from either restricted regions of the brain (focal epilepsy) or from widespread brain activation (generalised epilepsy). They present as stereotyped signs and symptoms that are generally determined by the site of increased electrical activity. Epilepsy contributes to over 0.5% of the total global disease burden and, as of 2017, the estimated global lifetime prevalence of epilepsy was 7.6 per 1000 persons, with an incidence rate of 61.44 per 100,000 person-years [1, 2]. It is associated with multiple ictal and interictal complications, which may arise from seizure-induced physical trauma, as well as disturbances to cardiorespiratory, metabolic, and neuropsychiatric states. Imminently life-threatening episodes, particularly status epilepticus and Sudden Unexplained Death in Epilepsy (SUDEP), may also occur in poorly controlled cases.

Current management approaches begin with anti-seizure medication (ASM) regimens. Around 51% of patients, however, do not achieve seizure freedom with the first ASM alone, and approximately 25–35% of patients progress to develop drug-resistant epilepsy (DRE) [3–5], which is defined as the failure to achieve seizure freedom despite two adequate trials of tolerated and appropriately chosen ASM regimens [6]. Resective or ablative epilepsy represents the greatest avenue for seizure control for DRE patients, although only 60% of patients who undergo surgical management achieve seizure freedom when observed at a 10-year follow-up [7]. Surgical management is also not a feasible option in all DRE patients, with some being unsuitable for surgery and others refusing surgical options due to associated risks. Few treatment options remain viable for these patients, with dietary therapies and invasive neuromodulation currently considered to be the two main alternative options for this population [8–10]. Non-invasive neuromodulation, thus, presents as a promising alternative or adjunctive treatment for greater seizure control, carrying significant advantages over both dietary and invasive neuromodulatory approaches to management due to its low-risk profile and non-invasive nature.

Non-invasive neuromodulation encompasses multiple modalities that stimulate either the brain or peripheral nerves. Repetitive transcranial magnetic stimulation (rTMS), transcranial direct current stimulation (tDCS), transcranial alternating current stimulation (tACS), and low-intensity focused ultrasound (LI-FUS) are methods used to directly and non-invasively target sites of seizure onset in the cerebral cortex [11]. The efficacy of rTMS was investigated in a recently published systematic review [12]. A significant reduction in seizure frequency of 72% and 78.9% was reported in a randomised double-blinded, sham-controlled trial and a randomised single-blinded non-controlled trial, respectively. This outcome, however, was not observed in the other six trials, which comprised four double-blinded and two unblinded randomised trials, five of which were controlled. Due to this and heterogeneity in trial methodologies, definitive conclusions about rTMS’s anti-seizure effects could not be drawn. On the other hand, a meta-analysis of eight randomised controlled trials investigating the anti-seizure properties of tDCS [13] reported a significant mean difference in seizure frequency between treatment and control groups after 4 weeks (MD =  − 4.72, 95% CI [− 6.80, − 2.65], *P* < 0.05) and 8 weeks (MD =  − 3.39, 95% CI [− 6.07, − 0.72], *P* < 0.05) of follow-up. A recent review [14] also found a significant decrease in overall seizure frequency in 14 of the 16 included randomised controlled trials, uncontrolled trials, and case series. There were, however, great variations in the degree of seizure frequency reduction and in the longevity of these suppressive effects between studies, limiting the review’s overall confidence in its conclusions. Regarding LI-FUS and tACS, studies evaluating their anti-seizure effects are fewer in number, and to the best of our knowledge, there are currently no systematic reviews with a focus on these interventions for DRE. A small pilot study investigating the safety of LI-FUS, however, reported no changes to neuropsychological status nor any detectable damage to targeted cerebral tissue (evaluated via post-resection histological analysis), offering promising data regarding the safety of LI-FUS [15].

Non-invasive *nerve* stimulation techniques—transcutaneous vagus nerve stimulation (tVNS) and trigeminal nerve stimulation (TNS)—also demonstrate anti-seizure potential. They aim to achieve this by stimulating peripheral nerve branches and indirectly modulating connected proximal cerebral regions that are implicated in seizure onset. Although no systematic reviews have explored the efficacy of TNS, a recent meta-analysis of tVNS [16] reported a significant mean difference in seizure frequency reduction between treatment and control groups (MD = −3.29, *P* = 0.03, 95% confidence interval (CI) −6.31 to −0.27,I^2^ = 10%) across three randomised control trials comprised of 280 patients. A 2021 systematic review of tVNS, however, reported mean seizure frequency reductions varying from 30 to 65% across the 10 studies it investigated [17]. As such, although promising, further clinical trials are necessitated to establish a more comprehensive and reliable understanding of these neuromodulatory approaches to epilepsy management.

Moreover, stimulation techniques, parameters, and other study methodologies appear to vary greatly between studies, making it difficult to establish clear and reliable study conclusions. Explorations into optimal neuromodulation protocol settings will help to inform future trial protocols and allow for greater consistency between future trials, enabling a greater capacity to compare, evaluate, and interpret study findings.

### Objectives

Non-invasive neuromodulation for the treatment of DRE remains in its infancy, and our understanding of its role in epilepsy management continues to evolve. This systematic review and meta-analysis aims to investigate the efficacy of rTMS, tDCS, tACS, LI-FUS, tVNS, and TNS for seizure reduction amongst patients diagnosed with DRE, with comparisons also being made between intervention types where applicable. Both immediate and long-term adverse events will be discussed in addition to this to offer greater insight into its use as a safe, viable, and reliable method of seizure control in the future. The study’s secondary aim will be to identify optimal stimulation parameters to better inform future clinical trial protocols and to maximise treatment efficacy in clinical applications.

## Methods

This protocol will adhere to the PRISMA-P Guidelines for the reporting of systematic review protocols [18] and the systematic review will adhere to PRISMA Guidelines for the reporting of systematic reviews ​​[19].

### Information sources

Relevant studies will be searched on PubMed, OVID Medline, OVID Embase, and Web of Science on 06/08/2023–10/08/2023 and 22/05/2024. Trial registries will also be searched to identify and trace associated studies reporting original outcome data. An updated search will be performed prior to final submission.

### Search strategy

The search criteria will be developed by SP and HS. Search strategies for all intervention types on PubMed will be supplied in Supplementary material 1. The same keywords, keyword combinations, and restrictions will be applied across all databases.

Restrictions will be placed to limit studies to those published in English between 01/01/1985 and 22/05/2024 and up until the date of the updated search, which will be performed prior to final submission. No restrictions will be placed on document type or publication status.

### Eligibility criteria

All studies included will be required to be written in English and published between 01/01/1985 to 22/05/2024 and up until the date of the updated search, which will be performed prior to final submission. Unpublished studies will not be sought.

#### Study design

Peer-reviewed randomised control trials, cohort studies and case-control studies will be included. Cross-sectional studies, ecological studies, case series/reports, conference abstracts, non-peer-reviewed studies, systematic reviews and meta-analyses will all be excluded.

#### Participants

Study populations will be required to include living human participants with DRE only. Studies with non-human studies, no description of DRE in human participants, in-patient participants, participants experiencing Status Epilepticus and a participant population including neonates and infants will be excluded from the review. Studies containing participants experiencing Epilepsia Partialis Continua, however, will be included.

#### Intervention

Study interventions must include one of rTMS, tDCS, tACS, LI-FUS, tVNS (either auricular or cervical) or TNS. Study interventions containing no brain or nerve stimulation techniques, multiple non-invasive stimulation techniques applied simultaneously or invasive brain and nerve stimulation techniques will not be included in the review.

#### Comparison

Studies with comparisons will be included if such comparison groups were given placebo/sham interventions, ASM regimens established prior to the study or no treatment at all. Studies with no controls or comparison group will also be included. Any study with healthy controls or non-living human controls will be excluded.

#### Outcome

All included studies will be required to report the primary outcome of “Change to seizure frequency”. This can be reported as either a Responder Rate or percentage change in seizure frequency. Studies will not have to report the secondary outcome (any adverse events reported by participants) to be included. Any study with no measure of seizure frequency (reported as either a Responder Rate or percentage change) will be excluded.

### Study selection

Studies will be independently screened on Covidence (Veritas Health Innovation, Melbourne, Australia) by SP and HS in two stages, with conflicts being resolved by a third reviewer, MZ. Stage 1 will involve screening studies by title and abstract, and Stage 2 will involve the full text screening of all studies passing Stage 1. All studies excluded in Stage 2 will be documented with a removal reason in Covidence, and attempts to contact authors of original studies in which the full text could not be accessed will be made. A study will be included for meta-analyses if it includes a control group (placebo, sham, treatment with anti-seizure medications, no treatment) and will be excluded if it has no controls, healthy controls, or non-living human controls.

### Data management/extraction

All studies that passed the second stage of screening (listed in Covidence) will be transferred to a spreadsheet. All items will be extracted manually and reported on the spreadsheet by SP and HS for each study. Data will be extracted from tables and text and, when needed, from graphs by two independent reviewers using the digital screen ruler ‘PlotDigitizer’. Attempts to contact authors of original studies will be made when important data is missing.

Extracted data items include:

Population:

- Sex
- Age
- Number of participants with each type of epilepsy
- Age of seizure onset
- Age of epilepsy diagnosis
- Seizure types
- Seizure syndrome
- For focal epilepsy: location(s) of seizure onset
- Electroencephalography findings
- Brain magnetic resonance imaging findings
- Seizure frequency during baseline period
- Anti-seizure medications/treatments taken before study
- Previous resective/ablative epilepsy surgery and region of surgery
- Current invasive stimulation

Method

- Number of human participants in study group
- Number of human participants in control group
- Number of groups
- Type of intervention administered
- Number of people excluded from reported data analyses
- Duration of treatment period
- Duration of interval between treatment periods
- Duration of each treatment sessions
- Intensity, frequency, pulse duration, pulse pattern, and number of pulses for each stimulation technique (may vary with type of intervention)
- Continuous or intermittent/discontinuous pulses/stimulation
- Type of coil/stimulator
- Location stimulation applied to
- Location of anatomical target
- Type of targeting used: none, routine EEG, high density EEG, anatomical only, high density EEG + MRI (navigated TMS, nTMS)
- Stimulation equipment/machine used
- Follow-up time after therapy

Primary Outcome(s)

- Primary endpoint – “Responder rate”: Percentage of participants with a 50% reduction in seizure frequency compared to baseline, as observed at the ‘end of treatment’ and the ‘end of follow-up’.
- Secondary endpoint – “Mean seizure reduction”: Absolute difference in seizure counts for baseline, as observed at the ‘end of treatment’ and the ‘end of follow-up’.

Secondary Outcome(s)

- Number of participants with seizure precipitation, headache, muscle twitch, dizziness, tinnitus, paraesthesia, and local irritation reported

### Data synthesis

A quantitative synthesis is planned if the included studies are sufficiently homogeneous. All data analyses will be conducted using *Stata* version 16.1 (StataCorp). The method of data synthesis for each outcome investigated in the review is specified below:

#### Primary outcome

“Responder rate” (primary endpoint) and “Mean seizure reduction” (secondary endpoint): A descriptive summary will be used to combine/compare the data. Quantitative data will be synthesised for stimulation techniques with sufficient data to do so via meta-analyses, which will be stratified by NIN modality type. In addition to this, comparisons will be made between NIN methods and control groups. For this, odds ratios will be estimated for the ‘Responder Rate’, and mean differences for ‘mean seizure reduction’ when there is sufficient control data to do so.

The pooled effect sizes for the “Responder rate” and corresponding odds ratios will be assessed using aggregate data. To mitigate the impact of small and heterogeneous study sets, pooled estimates will be calculated using a random-effects inverse-variance model with the Sidik-Jonkman estimator for between-study variance (τ2). A continuity correction of 0.5 will be applied only to studies with at least one zero cell.

For the pooled effect sizes of “Mean seizure reduction” and corresponding mean differences, individual participant data (IPD) will be used wherever available to ensure consistent estimation. Mean seizure reduction will be calculated as the change from baseline seizure frequency (follow-up count minus baseline count) for each participant prior to pooling. When IPD is not available, aggregate data will be analysed under the assumption that reported values represent the mean of individual patient-level reductions, unless explicitly stated otherwise. For studies reporting only the median and interquartile range of seizure frequency, we will convert quantiles to means and standard deviations under the assumption of normality.

When both seizure diary-recorded and clinician-reported seizure counts are available, the data from the seizure diary will be used to maximise accuracy. Outliers in individual seizure change data will be identified using distributional criteria, defined as values greater than three standard deviations from the mean or outside 1.5 times the interquartile range. Sensitivity analyses will be performed with and without identified outliers to evaluate their influence on the mean seizure reduction and the pooled meta-analytic estimate. If outliers substantially alter the results, both analyses (with and without outliers) will be reported for transparency.

A two-stage IPD meta-analysis will be performed, combining studies with IPD and those reporting only aggregate data, using a random-effects inverse-variance model with the Sidik-Jonkman two-step τ2 estimator.

Heterogeneity will be investigated via Q and I^2^, alongside visual inspection of forest plots and review of pooled effect size estimates with their 95% confidence intervals. A meta-regression will be performed in addition to this to assess whether study-level covariates significantly affect the effect size and to investigate any sources of heterogeneity. Adjusted R^2^ values will also be determined to assess the explanatory power of the variables included in the meta-regression.

Sensitivity analyses will include the exclusion of low-quality studies and leave-one-out analysis, where applicable. Low-quality studies are defined as studies scoring either a < 50% on the JBI Critical Appraisal Tool ‘Quasi-Experimental Studies’ checklist or a ‘High’ risk of bias score from the Cochrane Risk of Bias 2 Tool.

#### Secondary outcome

##### Percentage of participants reporting adverse events

A descriptive summary will be used to combine/compare this data. Reported adverse events will be categorised as occurring either “during treatment/stimulation” or “after treatment/stimulation”, as “serious or life-threatening” or “not serious or life-threatening”, and will be mapped into the following categories for each intervention: seizure precipitation, headache, muscle twitch, dizziness, tinnitus, paraesthesia, local irritation, and other notable adverse events. If sufficient data are available, an odds ratio will be calculated, where possible, to compare intervention and control groups.

Subgroup analyses will investigate the below groups:

- Type of epilepsy: focal, generalised
- Different stimulation protocol settings. This may include: duration of treatment, number of pulses, type of coil/stimulator, location of applied stimulation, and other key stimulation parameters as relevant to each modality.

All planned meta-regression and subgroup analyses will be exploratory in nature, and no formal claims will be made without replication.

#### Risk of bias assessment

Two reviewers will independently assess each study’s risk of bias, with discrepancies being resolved by a third reviewer. Quasi-experimental studies (non-randomised trials) will be evaluated via the JBI Critical Appraisal Tool for Quasi-Experimental Studies checklist. Randomised control trials will be evaluated by the Cochrane Risk of Bias 2 Tool (RoB 2), and randomised crossover trials will be assessed by the Cochrane Risk of Bias 2 Tool (RoB 2) for crossover trials.

#### Publication bias

A funnel plot, Egger’s test, Begg’s test, and Trim and Fill will be used to assess for publication bias.

#### Strength of evidence

Will be assessed via Grading of Recommendations Assessment, Development and Evaluation [20]

#### Protocol amendments

The protocol was originally registered in PROSPERO on 11/08/2023. The most recent amendment was submitted on 14/08/2024, with descriptions and justifications for all protocol amendments carefully documented in the PROSPERO database.

## Discussion

Non-invasive neuromodulation is emerging as a safe and effective treatment option for people with DRE; hence, there is increasing interest in the evidence base around it. While there have been systematic reviews on individual modalities, they are limited by their focus on individual techniques. These reviews typically use different methodologies; hence, comparisons between them and the modalities they focus on are challenging. This review, however, will integrate *all* relevant neuromodulation techniques to allow for comprehensive comparisons and evaluations of stimulation method efficacy and safety. It will also uniquely offer insight into optimal stimulation parameters to better inform the use of these methods in future trials and clinical applications.

Standard evidence-based practices will be used for the systematic review, including registering the protocol on the Prospero registry and following the PRISMA guidelines for reporting of systematic reviews. Major online databases of the scientific literature will be incorporated using the Covidence platform. Standard assessments of risk of bias and standardised statistical tools will be employed.

In operationalising the protocol, a number of observations were made and resulted in minor amendments to the protocol. With respect to the population, it was clarified that the intended study population was outpatient chronic treatment, rather than acute inpatient treatment of status epilepticus. With respect to the risk of bias assessment, the Cochrane risk of bias tool was preferred over the JBI tool, due to the significant number of RCTs noted on initial search results. Similarly, with respect to statistical analysis, the odds ratio was considered to be the most appropriate outcome measure, as opposed to relative risk, as it better accounts for the possible inclusion of case–control studies. All amendments to the protocol are carefully documented in the Prospero registry entry.

## Supplementary Information

Supplementary Material 1: Search terms.

## Data Availability

Data sharing is not applicable to this article as no datasets were generated or analysed during the current study.

## Abbreviations

ASM: Anti-seizure medications
DRE: Drug-resistant epilepsy
LI-FUS: Low intensity focused ultrasound
RoB: Risk of Bias
rTMS: Repetitive transcranial magnetic stimulation
SUDEP: Sudden unexpected death in epilepsy
tACS: Transcranial alternating current stimulation
tDCS: Transcranial direct current stimulation
TNS: Trigeminal nerve stimulation
tVNS: Transcutaneous vagus nerve stimulation

## References

[1] Fiest KM, Sauro KM, Wiebe S, Patten SB, Kwon C-S, Dykeman J, et al. Prevalence and incidence of epilepsy: a systematic review and meta-analysis of international studies. Neurology. 2017;88:296–303. 10.1212/WNL.0000000000003509. 27986877 10.1212/WNL.0000000000003509 PMC5272794

[2] World Health Organization. Epilepsy: a public health imperative. Geneva: World Health Organization; 2019.

[3] Chen Z, Brodie MJ, Liew D, Kwan P. Treatment outcomes in patients with newly diagnosed epilepsy treated with established and new antiepileptic drugs: a 30-year longitudinal cohort study. JAMA Neurol. 2018;75:279. 10.1001/jamaneurol.2017.3949. 29279892 10.1001/jamaneurol.2017.3949 PMC5885858

[4] Shankar R, Marston XL, Danielson V, Do Rego B, Lasagne R, Williams O, et al. Real-world evidence of epidemiology, patient characteristics, and mortality in people with drug-resistant epilepsy in the United Kingdom, 2011–2021. J Neurol. 2024;271:2473–83. 10.1007/s00415-023-12165-4. 38240828 10.1007/s00415-023-12165-4 PMC11055725

[5] Sultana B, Panzini M-A, Veilleux Carpentier A, Comtois J, Rioux B, Gore G, et al. Incidence and prevalence of drug-resistant epilepsy: a systematic review and meta-analysis. Neurology. 2021;96:805–17. 10.1212/WNL.0000000000011839. 33722992 10.1212/WNL.0000000000011839

[6] Kwan P, Arzimanoglou A, Berg AT, Brodie MJ, Allen Hauser W, Mathern G, et al. Definition of drug resistant epilepsy: consensus proposal by the ad hoc task force of the ILAE commission on therapeutic strategies. Epilepsia. 2010;51(6):1069–77. 10.1111/j.1528-1167.2009.02397.x. 19889013 10.1111/j.1528-1167.2009.02397.x

[7] Bjellvi J, Olsson I, Malmgren K, Wilbe Ramsay K. Epilepsy duration and seizure outcome in epilepsy surgery: a systematic review and meta-analysis. Neurology. 2019;93. 10.1212/WNL.0000000000007753. PMC6656653 31182508

[8] Alcala-Zermeno JL, Gregg NM, Starnes K, Mandrekar JN, Van Gompel JJ, Miller K, et al. Invasive neuromodulation for epilepsy: comparison of multiple approaches from a single center. Epilepsy Behav. 2022;137:108951. 10.1016/j.yebeh.2022.108951. 36327647 10.1016/j.yebeh.2022.108951 PMC9934010

[9] Lundstrom BN, Osman GM, Starnes K, Gregg NM, Simpson HD. Emerging approaches in neurostimulation for epilepsy. Curr Opin Neurol. 2023;36:69–76. 10.1097/WCO.0000000000001138. 36762660 10.1097/WCO.0000000000001138 PMC9992108

[10] Simpson HD, Schulze-Bonhage A, Cascino GD, Fisher RS, Jobst BC, Sperling MR, et al. Practical considerations in epilepsy neurostimulation. Epilepsia. 2022;63:2445–60. 10.1111/epi.17329. 35700144 10.1111/epi.17329 PMC9888395

[11] Starnes K, Schulze-Bonhage A, Lundstrom B. Noninvasive brain stimulation for epilepsy, in: Neurostimulation for Epilepsy. Elsevier, 2023 pp. 175–194. 10.1016/B978-0-323-91702-5.00012-8.

[12] Walton D, Spencer DC, Nevitt SJ, Michael BD. Transcranial magnetic stimulation for the treatment of epilepsy. Cochrane Database of Systematic Reviews 2021. 2021. 10.1002/14651858.CD011025.pub3. PMC8092469 33884611

[13] Hawas Y, Alkhawaldeh I, Alsalhi H, Matin R, Ibrahim G, Negida A. Transcranial direct current stimulation (tDCS) in treatment of refractory epilepsy: a systematic review and meta-analysis (P3–1.011). Neurology. 2024;102:6299. 10.1212/WNL.0000000000206380.

[14] Sudbrack-Oliveira P, Barbosa MZ, Thome-Souza S, Razza LB, Gallucci-Neto J, Da Costa Lane Valiengo L, et al. Transcranial direct current stimulation (tDCS) in the management of epilepsy: a systematic review. Seizure. 2021;86:85–95. 10.1016/j.seizure.2021.01.020. 33582584 10.1016/j.seizure.2021.01.020

[15] Stern JM, Spivak NM, Becerra SA, Kuhn TP, Korb AS, Kronemyer D, et al. Safety of focused ultrasound neuromodulation in humans with temporal lobe epilepsy. Brain Stimul. 2021;14:1022–31. 10.1016/j.brs.2021.06.003. 34198105 10.1016/j.brs.2021.06.003

[16] Wu K, Wang Z, Zhang Y, Yao J, Zhang Z. Transcutaneous vagus nerve stimulation for the treatment of drug-resistant epilepsy: a meta-analysis and systematic review. ANZ J Surg. 2020;90:467–71. 10.1111/ans.15681. 32052569 10.1111/ans.15681

[17] Lampros M, Vlachos N, Zigouris A, Voulgaris S, Alexiou GA. Transcutaneous vagus nerve stimulation (t-VNS) and epilepsy: a systematic review of the literature. Seizure. 2021;91:40–8. 10.1016/j.seizure.2021.05.017. 34090145 10.1016/j.seizure.2021.05.017

[18] Moher D, Shamseer L, Clarke M, Ghersi D, Liberati A, Petticrew M, et al. Preferred reporting items for systematic review and meta-analysis protocols (PRISMA-P) 2015 statement. Syst Rev. 2015;4:1. 10.1186/2046-4053-4-1. 25554246 10.1186/2046-4053-4-1 PMC4320440

[19] Page MJ, McKenzie JE, Bossuyt PM, Boutron I, Hoffmann TC, Mulrow CD, Shamseer L, Tetzlaff JM, Akl EA, Brennan SE, Chou R, Glanville J, Grimshaw JM, Hróbjartsson A, Lalu MM, Li T, Loder EW, Mayo-Wilson E, McDonald S, McGuinness LA, Stewart LA, Thomas J, Tricco AC, Welch VA, Whiting P, Moher D. The PRISMA 2020 statement: an updated guideline for reporting systematic reviews. BMJ. 2021;n71. 10.1136/bmj.n71.

[20] Brożek JL, Akl EA, Alonso-Coello P, Lang D, Jaeschke R, Williams JW, et al. Grading quality of evidence and strength of recommendations in clinical practice guidelines: part 1 of 3. an overview of the GRADE approach and grading quality of evidence about interventions. Allergy. 2009;64:669–77. 10.1111/j.1398-9995.2009.01973.x. 19210357 10.1111/j.1398-9995.2009.01973.x

