# Supplementary material for: Non-invasive neuromodulation for the treatment of drug-resistant epilepsy: Protocol for a systematic review and meta-analysis investigating efficacy, safety, and optimal stimulation parameters
Source: Syst Rev. 2025 Nov 6;14:214. doi: 10.1186/s13643-025-02981-2 (PMC12593945; doi:10.1186/s13643-025-02981-2)
Supplement: Supplementary file 1 — Supplementary Material 1: Search terms. [file 13643_2025_2981_MOESM1_ESM.pdf]

Supplemental Material 1 – PubMed Searches

rTMS

((Epilepsy) OR (Drug-Resistant Epilepsy) OR (Drug-Refractory Epilepsy) OR (DRE) OR (Medication-Resistant Epilepsy) OR (Medication-Refractory Epilepsy) OR (Medically Refractory Epilepsy) OR (MRE) OR (Pharmacologically Resistant Epilepsy) OR (Pharmacoresistant Epilepsy) OR (Focal Epilepsy) OR (Seizure) OR (Spontaneous Recurrent Seizures) OR (SRS) OR (Seizure Disorder)) AND ((Transcranial Magnetic Stimulation) OR (TMS) OR (Repetitive Transcranial Magnetic Stimulation) OR (Repetitive TMS) OR (rTMS) OR (Low Frequency Transcranial Magnetic Stimulation) OR (Low Frequency TMS))

tDCS

((Epilepsy) OR (Drug-Resistant Epilepsy) OR (Drug-Refractory Epilepsy) OR (DRE) OR (Medication-Resistant Epilepsy) OR (Medication-Refractory Epilepsy) OR (Medically Refractory Epilepsy) OR (MRE) OR (Pharmacologically Resistant Epilepsy) OR (Pharmacoresistant Epilepsy) OR (Focal Epilepsy) OR (Seizure) OR (Spontaneous Recurrent Seizures) OR (SRS) OR (Seizure Disorder)) AND ((Transcranial Direct Current Stimulation) OR (Transcranial DCS) OR (tDCS))

tACS

((Epilepsy) OR (Drug-Resistant Epilepsy) OR (Drug-Refractory Epilepsy) OR (DRE) OR (Medication-Resistant Epilepsy) OR (Medication-Refractory Epilepsy) OR (Medically Refractory Epilepsy) OR (MRE) OR (Pharmacologically Resistant Epilepsy) OR (Pharmacoresistant Epilepsy) OR (Focal Epilepsy) OR (Seizure) OR (Spontaneous Recurrent Seizures) OR (SRS) OR (Seizure Disorder)) AND ((Transcranial Alternating Current Stimulation) OR (Transcranial ACS) OR (tACS))

LI-FUS

((Epilepsy) OR (Drug-Resistant Epilepsy) OR (Drug-Refractory Epilepsy) OR (DRE) OR (Medication-Resistant Epilepsy) OR (Medication-Refractory Epilepsy) OR (Medically Refractory Epilepsy) OR (MRE) OR (Pharmacologically Resistant Epilepsy) OR (Pharmacoresistant Epilepsy) OR (Focal Epilepsy) OR (Seizure) OR (Spontaneous Recurrent Seizures) OR (SRS) OR (Seizure Disorder)) AND ((Low Intensity Focused Ultrasound) OR (Low Intensity FUS) OR (LI-FUS))

tVNS

((Epilepsy) OR (Drug-Resistant Epilepsy) OR (Drug-Refractory Epilepsy) OR (DRE) OR (Medication-Resistant Epilepsy) OR (Medication-Refractory Epilepsy) OR (Medically Refractory Epilepsy) OR (MRE) OR (Pharmacologically Resistant Epilepsy) OR (Pharmacoresistant Epilepsy) OR (Focal Epilepsy) OR (Seizure) OR (Spontaneous Recurrent Seizures) OR (SRS) OR (Seizure Disorder)) AND ((Transcutaneous Vagus Nerve Stimulation) OR (Transcutaneous VNS) OR (tVNS) OR (External Vagus Nerve Stimulation) OR (External VNS) OR (eVNS))

TNS

((Epilepsy) OR (Drug-Resistant Epilepsy) OR (Drug-Refractory Epilepsy) OR (DRE) OR (Medication-Resistant Epilepsy) OR (Medication-Refractory Epilepsy) OR (Medically Refractory Epilepsy) OR (MRE) OR (Pharmacologically Resistant Epilepsy) OR (Pharmacoresistant Epilepsy) OR (Focal Epilepsy) OR (Seizure) OR (Spontaneous Recurrent Seizures) OR (SRS) OR (Seizure Disorder)) AND ((Trigeminal Nerve Stimulation) OR (TNS) OR (External Trigeminal Nerve Stimulation) OR (External TNS) OR (eTNS))
